# Exploring the bacteriome in anthropophilic ticks: To investigate the vectors for diagnosis

**DOI:** 10.1371/journal.pone.0213384

**Published:** 2019-03-19

**Authors:** Aránzazu Portillo, Ana M. Palomar, María de Toro, Sonia Santibáñez, Paula Santibáñez, José A. Oteo

**Affiliations:** 1 Center for Rickettsiosis and Arthropod-Borne Diseases, Infectious Diseases Department, Hospital Universitario San Pedro-Center for Biomedical Research from La Rioja (CIBIR), Logroño, Spain; 2 Genomics and Bioinformatics Core Facility, CIBIR, Logroño, Spain; Onderstepoort Veterinary Institute, SOUTH AFRICA

## Abstract

**Objective:**

The aim of this study was to characterize the bacterial microbiome of hard ticks with affinity to bite humans in La Rioja (North of Spain).

**Methods:**

A total of 88 adult ticks (22 *Rhipicephalus sanguineus* sensu lato, 27 *Haemaphysalis punctata*, 30 *Dermacentor marginatus* and 9 *Ixodes ricinus*) and 120 *I*. *ricinus* nymphs (CRETAV collection, La Rioja, Spain), representing the main anthropophilic species in our environment, were subjected to a metagenomic analysis of the V3-V4 region of the 16S rRNA gene using an Illumina MiSeq platform. Data obtained with Greengenes database were refined with BLAST. Four groups of samples were defined, according to the four tick species.

**Results:**

Proteobacteria was the predominant phylum observed in all groups. Gammaproteobacteria was the most abundant class, followed by Alphaproteobacteria for *R*. *sanguineus*, *H*. *punctata* and *D*. *marginatus* but the relative abundance of reads for these classes was reversed for *I*. *ricinus*. This tick species showed more than 46% reads corresponding to ‘not assigned’ OTUs (Greengenes), and >97% of them corresponded to ‘*Candidatus* Midichloriaceae’ using BLAST. Within Rickettsiales, ‘*Candidatus* Midichloria’, *Rickettsia*, *Ehrlichia*, ‘*Candidatus* Neoehrlichia’ and *Wolbachia* were detected. *I*. *ricinus* was the most alpha-diverse species. Regarding beta-diversity, *I*. *ricinus* and *H*. *punctata* samples grouped according to their tick species but microbial communities of some *R*. *sanguineus* and *D*. *marginatus* specimens clustered together.

**Conclusions:**

The metagenomics approach seems useful to discover the spectrum of tick-related bacteria. More studies are needed to identify and differentiate bacterial species, and to improve the knowledge of tick-borne diseases in Spain.

## Introduction / Objective

The identification of microorganisms from biological samples has been dominated by the use of traditional culture-dependent methods and conventional molecular biology techniques (mostly polymerase chain reaction, PCR). The isolation of most tick-borne bacteria in synthetic media or in cell culture is difficult to obtain, and a high number of microbes remain uncultured. For the last two decades, the identification of *Rickettsia* spp. and other tick-associated pathogens has been mainly based on the use of specific PCR assays and sequence analysis [1,2]. Until recently, most studies focused on the detection of pathogens in vectors were able to detect a unique or a few microorganisms in a single assay. Metagenomic approaches, based on the development of the Next Generation Sequencing (NGS) techniques, and primary focused on the 16S rRNA study combined with bioinformatics tools, is revolutionizing the research in the fields of epidemiology and diagnosis of infectious diseases, among others, overcoming the limitation of detecting only one or few microorganisms at a time [3]. Metagenomic analysis can reveal the complexity of the microbiota of a given sample [4]. The number of pathogens associated with ticks has increased over the last years. Currently, there is a worldwide rising incidence of patients with a history of a tick-bite [5,6]. The importance of tick-borne diseases (TBDs) as a growing threat for public health has been recently underlined, and ‘*what is not sought*, *is not found*’ [7]. As ticks are able to transmit different microorganisms at one bite, it is necessary to be aware of possible co-infections. To investigate the microbial community composition harbored by ticks can facilitate the knowledge about the interactions among tick-associated microorganisms, the discovery of new uncultured microorganisms and subsequently, their implications as human pathogens.

Up to date, reports about metagenomics to investigate bacterial diversity of tick species are scarce. Our aim was to characterize the bacterial microbiome of hard ticks with affinity to bite humans in La Rioja (North of Spain).

## Materials and methods

### Tick samples

A total of 280 questing ticks (130 adults and 150 nymphs) belonging to the main species with affinity to bite humans in La Rioja (*Ixodes ricinus*, *Rhipicephalus sanguineus* sensu lato, *Dermacentor marginatus* and *Haemaphysalis punctata*) were selected from the -80°C freezer of the CRETAV collection (CIBIR, La Rioja, Spain) for the study of their bacterial profile. *Ixodes ricinus* is the most common arthropod vector of human diseases, and particularly nymphs of this species are the most frequent stage attacking humans in La Rioja [8]. Therefore, *I*. *ricinus* nymphs were also included in the study, in addition to adult specimens.

Ticks had been obtained from the field in La Rioja by flagging methods or by direct capture, either in urban habitats or in natural areas where outdoor activities are usually practiced, with the subsequent risk of infestation for humans (S1 Table). Specimens had been classified using taxonomic keys [9,10] and kept frozen at -80°C while still alive. Before DNA extraction, a half from every adult tick (longitudinally cut) was immediately frozen again at -80°C.

### DNA extraction

For DNA extraction, ticks were manipulated under sterile conditions in a Class II biosafety cabinet using cycles of UV light prior and between uses to prevent contamination. All the tools were also irradiated with UV light for at least 15 min. Sterile single-use instruments were used whenever possible. Non-disponsable material was sterilized between samples (e.g. forceps were rinsed in 70% ethanol and flamed). Ticks were surface-sterilized by immersion and shaking in 70% ethanol for two min. followed by rinsing twice in sterile deionized water (one min. each). All the solutions were sterile. Ticks were dried on autoclaved sterile filter paper, transferred to sterile petri dishes and cut into small fragments that were collected in sterile tubes. The DNA was extracted using DNeasy Blood and Tissue kit (Qiagen, Hilden, Germany), following the manufacturer’s instructions except for an overnight digestion and a final elution in 25 μL of warm (at 56°C) elution buffer. All the kit reagents had been previously tested for the absence of microorganisms using a pan-bacterial PCR [11]. Moreover, negative controls of extraction corresponding to extraction tubes without tick samples were included in parallel. DNA was quantified with a Qubit 3.0 fluorometer (Thermo Scientific) using Qubit dsDNA HS (High Sensitivity) assay kit. The quality of DNA was assessed by capillary electrophoresis with Fragment Analyzer (AATI) using Genomic DNA 50kb kit. DNA of enough quantity and quality for the NGS study was obtained from 88 adult ticks (22 *Rhipicephalus sanguineus* s.l., 27 *Haemaphysalis punctata*, 30 *Dermacentor marginatus* and 9 *Ixodes ricinus*) and 120 *I*. *ricinus* nymphs (in pools of ten individuals each) (S1 Table).

DNA extraction, preparation of PCR master mix, and amplification were performed in separate rooms to prevent contamination.

### 16S rRNA gene amplification, library preparation and sequencing

A total of 12.5 ng DNA per sample were used for the amplification step. Primers targeting the hypervariable V3 and V4 regions of 16S rRNA gene were used [12]. Amplified regions were purified and indexed with Nextera XT Index kit (Illumina). The library quality was assessed on a Qubit 3.0 Fluorometer (Thermo Scientific) and Fragment Analyzer (AATI) using dsDNA reagent (35-5000bp) kit. Paired-end 300 bp sequences were obtained on an Illumina MiSeq platform.

### Sequence processing and analysis

Quality controls of raw reads were carried out with FastQC software [13], and trimmed with the Trimmomatic software [14]. The V3-V4 amplified region (550–580 bp) was reconstructed through paired reads according the Quantitative Insights Into Microbial Ecology (QIIME) protocol (v1.9.1) [15]. Operational Taxonomic Units (OTUs) were defined as sequences with at least 97% similarity versus Greengenes database [16] using UClust clustering algorithm [17] and following the open-reference method described by QIIME [18]. OTUs with <0.01% relative abundance of the total read counts on a per-sample basis were removed (spurious and chimeric reads). Data were refined with BLAST tool against GenBank database using the consensus sequence from each OTU [19].

Four groups of samples were defined, according to the four tick species. Rarefaction curves were calculated prior to all analytical techniques in order to assess species richness from the samples. OTU abundance was normalized by Cumulative Sum Scaling (CSS) method with metagenomeSeq software [20] and barplots were constructed.

### Statistical analysis

Alpha diversity and relative evenness of communities’ analyses were calculated by Chao1, Fisher, Margalef, Observed OTUs, Phylogenetic diversity (PD) whole tree, Shannon, Simpson, and Singles indexes with QIIME. Similarity distance matrixes between species groups were calculated following Bray-Curtis, Weighted Unifrac and Unweighted Unifrac beta-diversity metrics. Principal Coordinate Analysis (PCoA) and Hierarchical Clustering Dendrograms (UPGMA) for each beta-diversity metric were drawn to visualize sample groupings. The Kruskall-Wallis (KW) test was calculated to study significant differences between species groups. Analysis was also performed with MicrobiomeAnalyst software [21].

## Results

A total of 19,977,253 read counts (average counts per sample = 201,790) and 227 OTUs were observed. The rarefaction curves reached a plateau, demonstrating that bacterial diversity had been satisfactorily detected for all samples (S1–S4 Figs).

**Fig 1:**
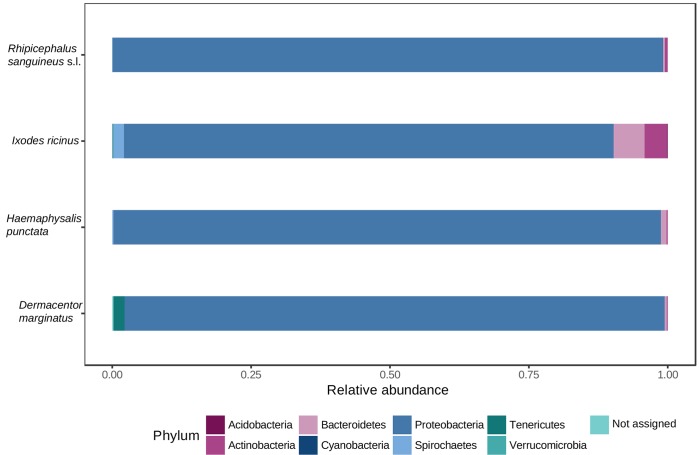
Phyla-level relative abundance of reads for each tick species analyzed. The histograms show the portion of MiSeq 16S rRNA gene sequences assigned to each phylum.

Proteobacteria was the dominant phylum in all tick species (S2 Table, Fig 1). Phyla Bacteroidetes, Actinobacteria, Acidobacteria, Tenericutes, Cyanobacteria, Verrucromicrobia and Spirochaetes were also observed in all groups (S2 Table, Fig 1).

At class level, Gammaproteobacteria and Alphaproteobacteria represented more than 82% of abundance of reads for the four tick species. Gammaproteobacteria was the most abundant class, followed by Alphaproteobacteria for *R*. *sanguineus* (95.25% and 3.65%), *H*. *punctata* (92.89% and 5.13%) and *D*. *marginatus* (80.92% and 15.90%). These percentages of relative abundance of reads were different for *I*. *ricinus*, in which predominated Alphaproteobacteria (70.41%) followed by Gammaproteobacteria (12.56%) (S2 Table). For Gammaproteobacteria, statistically significant differences (False Discovery Rate, FDR<0.05, calculated by the Kruskall-Wallis test) were found when *I*. *ricinus* was compared *vs*. *R*. *sanguineus* (FDR = 1.168e^-10^), *vs*. *H*. *punctata* (FDR = 1.229e^-11^), and *vs*. *D*. *marginatus* (FDR = 1.132e^-7^). Alphaproteobacteria showed significant differences between *D*. *marginatus* and *H*. *punctata* (FDR = 0.034), *D*. *marginatus* and *I*. *ricinus* (FDR = 0.020), *D*. *marginatus* and *R*. *sanguineus* (FDR = 0.008), *H*. *punctata* and *I*. *ricinus* (FDR = 0.492e^-3^), and *R*. *sanguineus* and *I*. *ricinus* (FDR = 0.732e^-3^) (S3 Table).

**Fig 2:**
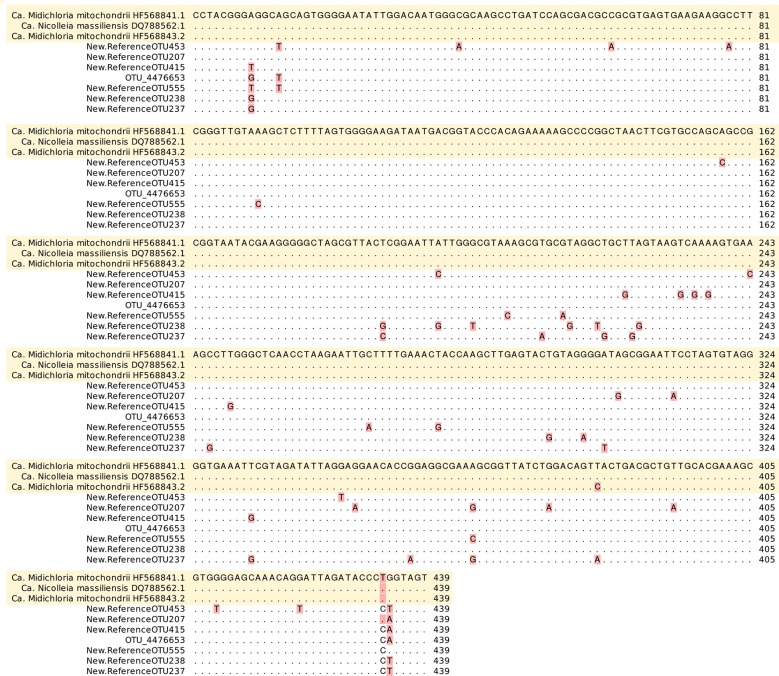
Nucleotide alignment of ‘*Candidatus* Midichloriaceae’ partial 16S rRNA references (according to BLAST) versus closed undefined OTUs (according to Greengenes database).

At least 23 orders were present (S2 Table). At family level, Coxiellaceae was the most abundant one for *D*. *marginatus* (79.96%), *H*. *punctata* (92.76%) and *R*. *sanguineus* (94.73%) but not for *I*. *ricinus* (0.77%). Abundance of reads for Coxiellaceae showed significant differences between *D*. *marginatus* and *I*. *ricinus* (FDR = 2.267e^-7^), *H*. *punctata* and *I*. *ricinus* (FDR = 2.049e^-13^), and *R*. *sanguineus* and *I*. *ricinus* (FDR = 1.635e^-10^) (S3 Table). At this level, *I*. *ricinus* showed the highest percentage (46.48%) corresponding to not assigned OTUs against Greengenes database (S2 Table). From them, 97.94% of reads (seven undefined OTUs according to Greengenes) showed maximum similarity with ‘*Candidatus* Midichloriaceae’ using BLAST (Fig 2).

Other detected families (>3%) were assigned to Rickettsiaceae (14.07 and 6.14%) for *D*. *marginatus* and *I*. *ricinus*; Pseudomonaceae (7.85%) and Oxalobacteraceae (3.55%) for *I*. *ricinus*; and Sphingomonadaceae (11.52% and 3.87%) for *I*. *ricinus* and *H*. *punctata*, respectively (S2 Table).

Within *I*. *ricinus*, sequences belonging to ‘*Candidatus* Midichloriaceae’ showed the highest identity with endosymbionts such as ‘*Candidatus* Midichloria mitochondrii’ or ‘*Candidatus* Nicolleia massiliensis’, according to BLAST analysis. They were prevalent in female samples (44.07–99.33%) but not in male specimens (0.57%), in which Pseudomonadaceae (60.19%) and Nocardiaceae (13.57%) were dominant (S4 Table).

Sequences assigned to ‘*Candidatus* Midichloriaceae’ using BLAST (that corresponded to not assigned OTUs against Greengenes) appeared in all *I*. *ricinus* nymph pools, with relative abundance of reads that ranged from 2.01 to 52.02% depending on the sample (S5 Table).

Within order Rickettsiales (17.26% abundance of reads), genera *‘Candidatus* Midichloria’, *Rickettsia*, *Ehrlichia*, *Anaplasma* and *Wolbachia* were found. However, *Anaplasma* sequences corresponded to ‘*Candidatus* Neoehrlichia mikurensis’ using BLAST (GenBank accession number KU535862). *Rickettsia* was the most abundant genus for *D*. *marginatus* and *R*. *sanguineus*, showing significant differences between *D*. *marginatus* and *R*. *sanguineus* (FDR = 0.002), *D*. *marginatus* and *I*. *ricinus* (FDR = 2.580e^-5^), and *D*. *marginatus* and *H*. *punctata* (FDR = 1.8694e^-6^). *Ehrlichia* was the most represented genus in *H*. *punctata*, but no significant differences were observed when comparing tick species in pairs. ‘*Ca*. Midichloria’ was the most abundant in *I*. *ricinus*. *Wolbachia* and ‘*Ca*. Neoehrlichia’ were more prevalent in *I*. *ricinus* than in the remaining groups. Significant differences for *Wolbachia* were observed between *I*. *ricinus* and *D*. *marginatus* (FDR = 0.109e^-3^); and for ‘*Ca*. Neoehrlichia’, between and *I*. *ricinus* and *D*. *marginatus* (FDR = 0.238e^-3^) and between *I*. *ricinus* and *H*. *punctata* (FDR = 0.007) (Table 1; S3 Table).

**Table 1:** Percentages of relative abundance of reads for genera within order Rickettsiales for each tick species (also by sex and stage when available) according to BLAST analysis.

Tick species	Rickettsiales				
	*‘Ca*. Midichloria’	*‘Ca*. Neoehrlichia’	*Ehrlichia*	*Rickettsia*	*Wolbachia*
*I*. *ricinus*	85.011	3.526	ND	1.400	10.063
Female	65.916	0.001	ND	0.010	ND
Male	0.007	ND	ND	ND	0.002
Nymphs	19.089	3.525	ND	1.390	10.061
*H*. *punctata*	0.976	0.018	96.697	2.059	0.251
Female	0.242	ND	0.036	1.397	ND
Male	0.734	0.018	96.661	0.662	0.251
*D*. *marginatus*	0.045	ND	0.099	99.955	*
Female	0.042	ND	0.099	69.057	ND
Male	0.003	ND	ND	30.897	*
*R*. *sanguineus* s.l.	1.402	0.020	0.002	98.530	0.047
Female	0.874	0.011	0.002	97.745	0.027
Male	0.528	0.009	ND	0.785	0.020

*Relative abundance of reads lower than 0.001%.

ND: not detected.

I.: Ixodes; H.: Haemaphysalis; D.: Dermacentor; R.: Rhipicephalus.

Bacteria belonging to the order Borreliales were minority (0.52% abundance of reads). Specifically, *Borrelia* spp. belonging to *B*. *burgdorferi* sensu lato (*B*. *garinii*) and relapsing fever group (*B*. *miyamotoi*) were detected (66.79% and 33.21%, respectively). *B*. *garinii* was mainly found in *I*. *ricinus* and it was less frequently detected in *R*. *sanguineus*, whereas *B*. *miyamotoi* showed the highest relative abundance of reads in *I*. *ricinus* followed by *H*. *punctata*. The joint presence of Rickettsiales and Borreliales was observed in *I*. *ricinus* as an example of potential source of human co-infections. Thus, female *I*. *ricinus* harboured *Rickettsia* with ‘*Ca*. Neoehrlichia’ or with *Borrelia* or with both genera.

Using BLAST, Entomoplasmatales (0.53% abundance of reads) appeared in all species, with predominance of *Spiroplasma* spp. (class Mollicutes) in *D*. *marginatus* (2.00%).

**Fig 3:**
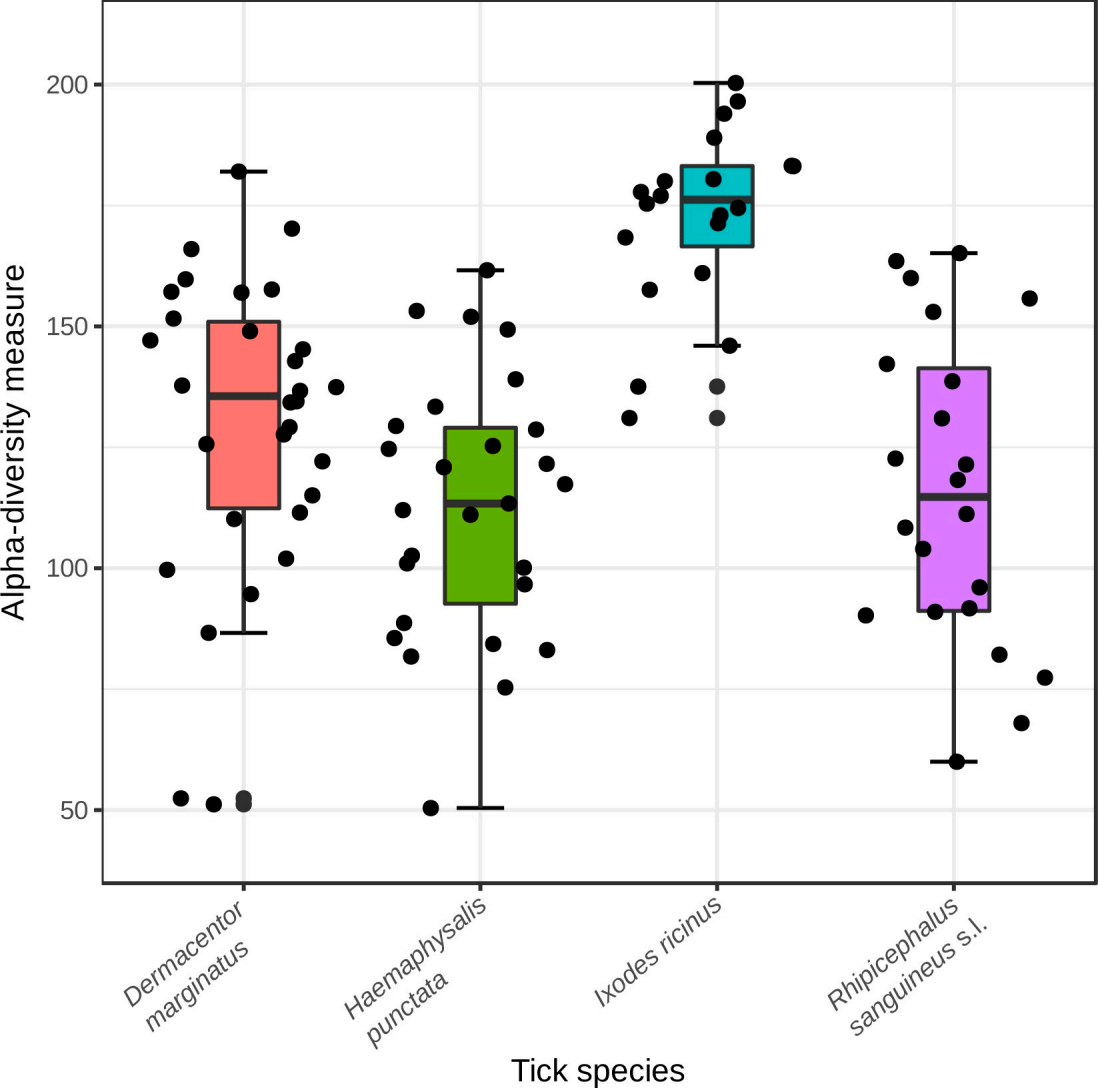
Chao1 alpha diversity index showing differences among tick species.

According to alpha-diversity measures, the mean alpha diversity was greater for *I*. *ricinus*, followed by *D*. *marginatus*, *R*. *sanguineus* and *H*. *punctata*. Differences in alpha diversity between *I*. *ricinus* and *D*. *marginatus*, between *I*. *ricinus* and *H*. *punctata* and between *I*. *ricinus* and *R*. *sanguineus* were statistically significant (p<0.01) using all but Singles index. The highest standard deviation of the mean appeared in *R*. *sanguineus* for all but Shannon, Simpson and Singles indexes, which showed the highest standard deviation of the mean in *I*. *ricinus* (Table 2). Group differences using Chao1 are showed in Fig 3.

**Table 2:** Compared values (mean and standard deviation) of alpha diversity indexes for tick species.

Index values		*I*. *ricinus*	*H*. *punctata*	*R*. *sanguineus* s.l.	*D*. *marginatus*
Chao1	**mean**	172.860	112.686	115.990	129.805
	**std**	18.502	26.982	32.106	31.377
Fisher	**mean**	18.543	10.312	10.430	11.894
	**std**	2.818	2.747	3.825	3.370
Margalef	**mean**	13.805	8.199	8.276	9.335
	**std**	1.776	2.021	2.752	2.419
Observed OTUs	**mean**	163.200	99.704	101.636	114.467
	**std**	19.579	26.159	33.894	29.688
PD whole tree	**mean**	14.642	9.881	10.732	11.006
	**std**	1.605	2.158	2.509	2.329
Shannon	**mean**	3.381	0.529	0.453	1.060
	**std**	1.653	0.498	0.426	0.582
Simpson	**mean**	0.698	0.114	0.096	0.301
	**std**	0.295	0.131	0.117	0.192
Singles	**mean**	12.750	15.481	16.955	16.267
	**std**	6.995	5.352	5.085	5.058

std: standard deviation; *I*.: *Ixodes; H*.: *Haemaphysalis; R*.: *Rhipicephalus;* s.l.: sensu lato; *D*.: *Dermacentor*.

**Fig 4:**
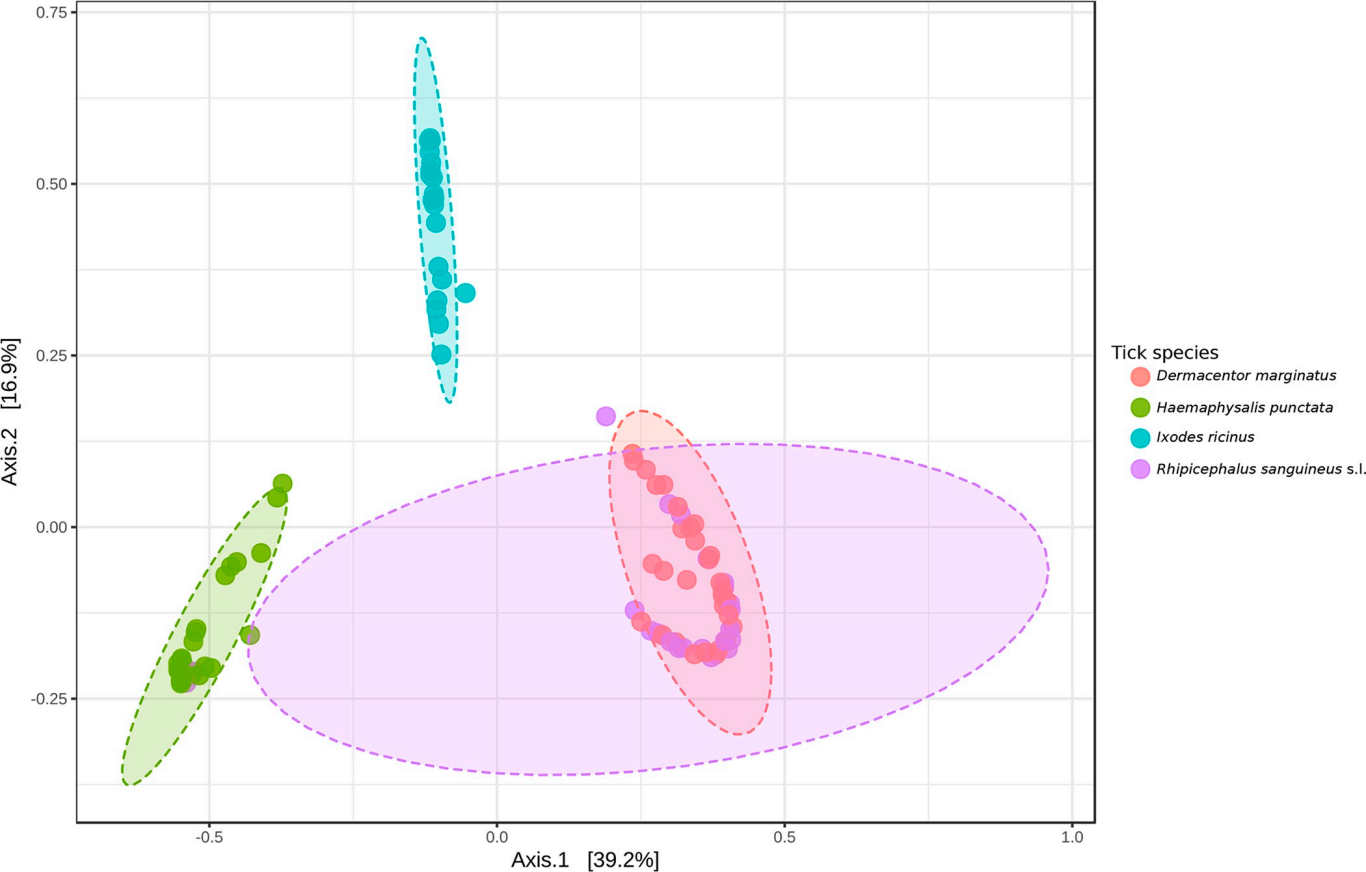
Principal Component Analysis (PCoA) generated among groups at genus level using Weighted UniFrac metric (a measure of differences in bacterial community structure).

Regarding beta diversity metrics (distance measure), using PCoA with Bray-Curtis or Weighted Unifrac distance index and Analysis of Group Similarities (ANOSIM) method at genus level, *I*. *ricinus* and *H*. *punctata* samples gathered according to their tick species. On the contrary, microbial communities of several specimens of *R*. *sanguineus* and *D*. *marginatus* groups clustered together, suggesting profile similarity (Fig 4).

**Fig 5:**
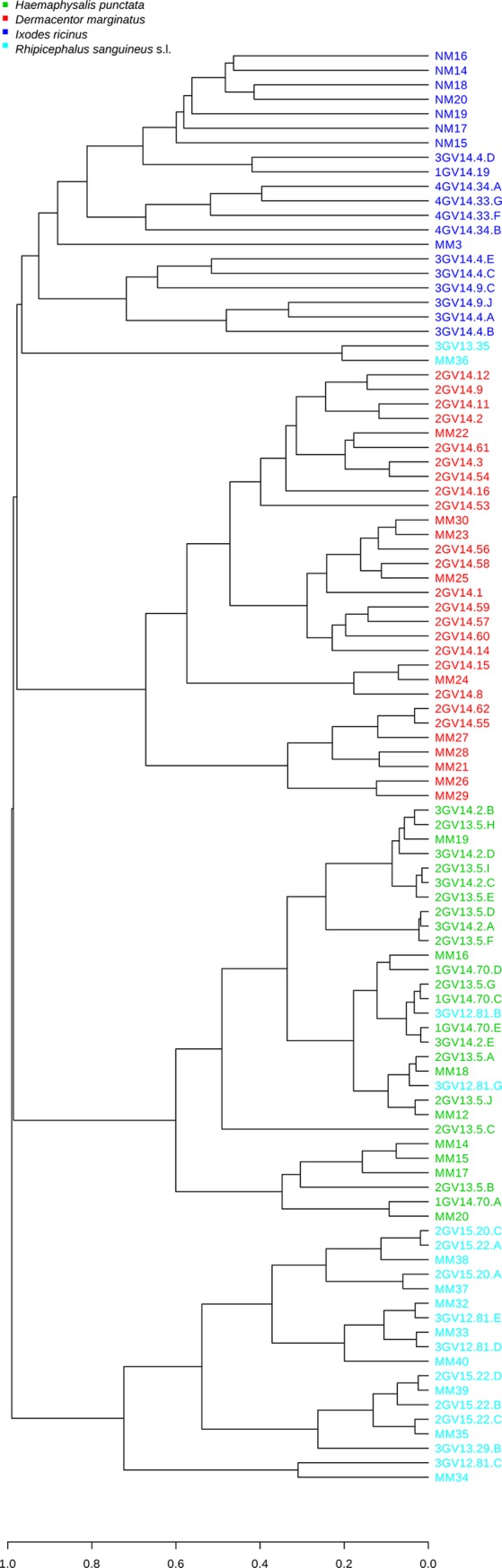
Cluster dendrogram generated among samples at OTU level using Bray Curtis distance index.

At OTU level, the best correlation between samples and tick species was showed using Bray-Curtis index. All samples grouped according to their tick species, except four *R*. *sanguineus* specimens that clustered within *H*. *punctata* (n = 2) or *I*. *ricinus* (n = 2) (Fig 5).

With respect to the analysis of differential abundance of reads, 30 OTUs were significantly present in a group and not in others (p<0.01): *Rickettsia* (2), *Coxiella* endosymbionts (18), *Spiroplasma* (2), *Ehrlichia* (1), *Pedobacter* (1), *Pseudomonadaceae* (1), *Sphingomonas wittichii* (1), *Spirosoma* (1) and 3 ‘not assigned’ OTUs (according to Greengenes) whose sequences showed 91% of maximum identity with *Coxiella* endosymbionts or with *Rickettsia* spp., according to BLAST.

## Discussion

Many TBDs have been recognized for the first time in the last few years, and emerging tick-borne pathogens are being detected [3,22–24]. Not only the clinical observation but also the application of new diagnostic methods (based on culture and molecular biology assays) has contributed to this progress [3]. Nevertheless, TBDs are dangerously expanding and they constitute underestimated causes of human illness worldwide [5]. The implementation of NGS platforms aimed to diagnosis is being developed, although reports about the contribution of this technique to the clinical diagnostic of TBDs are sporadic [25]. Herein, the bacteriome of tick species with affinity to bite humans was analysed using the 16S metagenomic approach to investigate tick-related microorganisms and to improve the diagnosis of TBDs, particularly in cases with unknown etiologic agents.

Data generated with NGS studies for tick microbiome characterization allow us to delve into microorganism interactions [26]. As reported by Estrada-Peña and Cabezas-Cruz (2019), recent findings about the tick microbiome are driving to a change of paradigm: ‘*most bacteria found in tick microbiome are fundamental for tick biological processes*’ [27]. We agree with that statement, although the ‘traditional’ point of view should not be forgotten. We have learned throughout history that microorganisms first detected in ticks, and for a long time considered non-pathogenic to humans, have been later implicated in human diseases (e.g. *Rickettsia parkeri*), even though some of them do not fulfil Koch's Postulates (e.g. ‘*Ca*. N mikurensis’, a not-yet-cultivated bacterium) [3,28–31]. The finding of an infectious agent in a vector could enable its involvement in human pathology, especially if repeatedly detected. Metagenomics can allow the identification of microorganisms carried by arthropod vectors in people with suspected TBDs, thus contributing to the etiological diagnostics. Clinicians should consider that infection with multiple TBDs is possible, especially in tick-endemic areas. In cases of co-infection with more than one pathogen the clinical symptoms may be longer and more severe than expected, and the diagnosis can be even more difficult [32,33]. Data analysis obtained from NGS methods can be promising for the simultaneous detection of tick-borne pathogens in patients suffering TBDs of unknown aetiology.

In our study, expected tick-associated bacteria (*Borrelia*, *Rickettsia*, *Coxiella*, *Spiroplasma*, *Ehrlichia*, *‘Ca*. Neoehrlichia’, *Wolbachia* and ‘*Ca*. Midichloria’) were found, as previously reported by other authors [34–38]. Other bacteria genera, associated to soil, water, plants, vertebrates or arthropods, and never reported to be related with TBDs were also identified herein: *Acinetobacter*, *Agrobacterium*, *Arthrobacter*, *Bosea*, *Bradyrhizobium*, *Brevundimonas*, *Burkholderia*, *Chryseobacterium*, *Comamonas*, *Devosia*, *Erwinia*, *Flavobacterium*, *Hymenobacter*, *Janthinobacterium*, *Kineococcus*, *Luteibacter*, *Luteolibacter*, *Methylibium*, *Methylobacterium*, *Methylopila*, *Mycobacterium*, *Mycoplana*, *Novosphingobium*, *Ochrobactrum*, *Paracoccus*, *Patulibacter*, *Pedobacter*, *Phyllobacterium*, *Pseudomonas*, *Rathayibacter*, *Rhizobium*, *Rhodobacter*, *Rhodococcus*, *Rhodoferax*, *Rubellimicrobium*, *Saccharothrix*, *Salinibacterium*, *Sphingomonas*, *Spirosoma*, *Stenotrophomonas*, *Streptomyces*, *Terriglobus* and *Williamsia*. Our findings suggest that these bacteria may be acquired from the environment. A recent study also recorded 15 of these genera associated to *I*. *ricinus* ticks collected from the field in France: *Arthrobacter*, *Bosea*, *Burkholderia*, *Devosia*, *Kineococcus*, *Luteibacter*, *Luteolibacter*, *Mycobacterium*, *Patulibacter*, *Pedobacter*, *Phyllobacterium*, *Spirosoma*, *Stenotrophomonas*, *Terriglobus* and *Williamsia* [39]. In addition, non-characterized bacteria whose pathogenicity remains unelucidated were detected based on the V3-V4 region of the 16S rRNA. More than 46% of ‘not assigned’ OTUs were found in *I*. *ricinus*, according to Greengenes. This database has been the preferred one for taxonomic classification due to its discrimination power at species level [40], but the gap for recently discovered bacteria is a weak point. When these OTU sequences were analysed with BLAST (GenBank database), they showed correspondence with ‘*Ca*. M. mitochondrii’, an endosymbiont belonging to the order Rickettsiales [41]. In addition, within the family Coxiellaceae (Greengenes), *Coxiella* endosymbionts were identified through GenBank sequence analysis, showing again the limitation of Greengenes as a referral database for the study of tick-associated bacteria. The same occurred with *Spiroplasma* spp. (order Entomoplasmatales), symbionts associated with ticks and other arthropods, and whose potential pathogenicity is discussed [42–44].

Bacteria corresponding to genus *Wolbachia* were also detected in our samples. *Wolbachia* spp. are obligate intracellular endosymbionts of arthropods and nematodes. There is evidence about the capacity of these bacteria to affect biology, physiology, immunity, ecology and evolution and reproduction of the hosts, and to influence other infectious diseases due to viruses, protozoa and filariae [42]. The co-occurrence of these microorganisms considered endosymbionts can constitute a valuable research field of future studies because the viability of ticks, or even of the pathogens that ticks are able to transmit, may depend on these endosymbionts.

According to our data, other examples of OTUs that could be better identified using BLAST were those that matched with *Anaplasma*, Borreliaceae and Entomoplasmatales. However, the identification was not possible for other ‘not assigned’ OTUs that showed 91% identity (the highest) with known sequences of *Rickettsia* spp. or *Coxiella* endosymbionts. These findings can be useful for a future targeted search of unknown bacteria associated with ticks, and their potential implications for human health.

According to our results, the composition of the microbiota of ticks was affected by sex and geography, as previously reported [45]. For instance, on the one hand, *H*. *punctata* males from our study showed higher relative abundance of reads for Rickettsiales than females of the species or other tick species. This pattern could be explained by different host preferences between males and females and/or influence of host hormones and/or higher adaptive capacity of the microorganism to the tick and/or relationships between tick microorganisms, among other factors. Nevertheless, our data refer to the abundance of reads but not to prevalence, and a bias may have occurred since females generally have much more of the endosymbiont than males. On the other hand, *D*. *marginatus* and *R*. *sanguineus* showed overlapping PCoA plots, maybe because specimens of both species were collected from the same site (Villalba de Rioja). There is preliminary evidence that ticks that are geographically close share microbes [45].

Of particular interest is our observation of the highest *I*. *ricinus* alpha diversity over the other tick species analyzed herein. The generalist behavior in host choice of *I*. *ricinus* could have played a major role in the great variability of this tick-associated microbiota. Nearly all the life cycle of this tick species is spent in the surface layers of soil or forest litter where environmental conditions influence its development. *I*. *ricinus* is the primary vector of a wide variety of pathogens with considerable impact on human and animal health [46]. Contrary to experiments that have demonstrated higher mortality rates of *R*. *sanguineus* infected with *Rickettsia conorii* than non-infected when exposed at low or high temperature [47], *I*. *ricinus* is a tick with potential to adapt to new climates as they change [48].

Unfortunately, the comparison of data between studies that evaluate tick microbiomes is complex since every research team is focused on different research interests. Variations in techniques, target regions of the 16S rRNA gene, reference taxonomic databases or source of tick samples may hinder comparisons. As an example, relevant information about the ecology of tick-associated microorganisms in ticks and in voles from a French area has been recently published [39]. However, our reads from *Coxiella* endosymbionts could not be accurately compared to those obtained by Estrada-Peña et al. (2018) due to differences in length of reads (V3-V4 *vs*. V4 region). A review of NGS strategies for the study of the microbiome of ticks shows an updated view of the current scene [49]. As the authors conclude, further studies aimed to assess the influences of the environments, the hosts or the ticks themselves on the diversity of the tick microbiomes are required. According to the authors, bacteriome tick findings must be completed with new ones focused on viruses and eukarya in ticks [49]. Herein, a picture of bacteriome of ticks in a certain environment is showed, although ticks also harbour viruses, protozoa, fungi, helminths, etc. [50] and plenty of questions remain unresolved. The technique has difficulties and possible bias due to: storage of samples, DNA extraction method, reagents contamination, amplified 16S rRNA regions, updating and maintenance of curated sequences by reference databases or multiple repeated partial sequences of GenBank database, among others. However, the metagenomic approach seems useful to discover the spectrum of bacteria carried by ticks. More studies are needed to identify and differentiate bacterial species, and to improve the knowledge of TBDs in Spain.

## Supporting information

S1 Table Data about tick species, sex (or stage) of ticks, sampling year, sites of sampling, coordinates, habitats and sampling methods of the specimens included in this study. (DOC)

S2 Table Bacterial taxa found in samples. Each sheet in the file describes share of taxa for each taxonomic level: phylum, class, order, family and genus. Taxonomic level is given on the sheet name.
(XLS)

S3 Table OTUs with statistically significant (p<0.05) abundance of reads at class, family and genus. Each sheet in the file describes comparisons between tick species for each taxonomic level: class, family and genus. Additionally, comparisons of male vs. female and nymphs vs. adults were performed for *I*. *ricinus* samples. This analysis was performed with MicrobiomeAnalyst <https://www.microbiomeanalyst.ca/> using a univariate analysis by the non-parametric Kruskall-Wallis test.
(XLSX)

S4 Table Family-level relative abundance of reads per sample for adult *I*. *ricinus* specimens. (XLSX)

S5 Table Family-level relative abundance of reads per sample for *I*. *ricinus* nymph pools. (XLSX)

S1 Fig Rarefaction curves of Observed OTUs richness for each sample. (TIFF)

S2 Fig This is the legend for S1 Fig. Sample IDs included in this study.
(TIF)

S3 Fig Rarefaction curves of Observed OTUs richness for each group (tick species). (TIFF)

S4 Fig This is the legend for S3 Fig. Sample groups (tick species) included in this study.
(TIF)

## References

[1] Portillo A, de Sousa R, Santibáñez S, Duarte A, Edouard S, Fonseca IP, et al Guidelines for the Detection of *Rickettsia* spp. Vector Borne Zoonotic Dis. 2017; 17:23–32. 10.1089/vbz.2016.1966 28055574

[2] Brouqui P, Bacellar F, Baranton G, Birtles RJ, Bjoërsdorff A, Blanco JR, et al Guidelines for the diagnosis of tick-borne bacterial diseases in Europe. Clin Microbiol Infect. 2004; 10:1108–32. 10.1111/j.1469-0691.2004.01019.x 15606643

[3] Portillo A, Oteo JA. New tools, new tick-borne pathogens? World J Clin Infect Dis. 2015; 5: 51–4.

[4] Lynch SV, Pedersen O. The Human Intestinal Microbiome in Health and Disease. N Engl J Med. 2016; 375:2369–79. 10.1056/NEJMra1600266 27974040

[5] Paules CI, Marston HD, Bloom ME, Fauci AS. Tickborne Diseases—Confronting a Growing Threat. N Engl J Med. 2018; 10.1056/NEJMp1807870 30044925

[6] Rosenberg R, Lindsey NP, Fischer M, Gregory CJ, Hinckley AF, Mead PS, et al Vital Signs: Trends in Reported Vectorborne Disease Cases—United States and Territories, 2004–2016. MMWR Morb Mortal Wkly Rep. 2018; 67:496–501. 10.15585/mmwr.mm6717e1 29723166 PMC5933869

[7] Oteo JA, Palomar AM. Crimean-Congo haemorrhagic fever: "What is not sought is not found". Med Clin (Barc). 2018; 150:266–7. 28867333 10.1016/j.medcli.2017.07.003

[8] Palomar AM. Role of birds in dispersal of ticks and their microorganisms. 2017. (PhD Thesis). https://dialnet.unirioja.es/descarga/tesis/122702.pdf

[9] Manilla G. Fauna D'Italia. Acari Ixodida, pp. 1–280. Calderini, Bologna; 1999.

[10] Estrada-Peña A, Bouattour A, Camicas JL, Walker AR. A guide to identification of species: ticks of domestic animals in Mediterranean region. London, UK; 2004.

[11] Black WC, Piesman J. Phylogeny of hard and soft tick taxa (Acari: ixodida) based on mitochondrial 16S rDNA sequences. Proc Natl Acad Sci USA. 1994; 91:10034–8. 7937832 10.1073/pnas.91.21.10034 PMC44952

[12] Klindworth A, Pruesse E, Schweer T, Peplies J, Quast C, Horn M, et al Evaluation of general 16S ribosomal RNA gene PCR primers for classical and next-generation sequencing-based diversity studies. Nucleic Acids Res. 2013; 41:e1 10.1093/nar/gks808 22933715 PMC3592464

[13] Babraham Bioinformatics—FastQC A quality control tool for high throughput sequence data. http://www.bioinformatics.babraham.ac.uk/projects/fastqc/

[14] Bolger AM, Lohse M, Usadel B. Trimmomatic: A flexible trimmer for Illumina Sequence Data. Bioinformatics. 2014; 30:2114–20. 10.1093/bioinformatics/btu170 24695404 PMC4103590

[15] Caporaso JG, Kuczynski J, Stombaugh J, Bittinger K, Bushman FD, Costello EK, et al QIIME allows analysis of high-throughput community sequencing data. Nat Methods. 2010; 7:335–6. 10.1038/nmeth.f.303 20383131 PMC3156573

[16] DeSantis TZ, Hugenholtz P, Larsen N, Rojas M, Brodie EL, Keller K, et al Greengenes, a chimera-checked 16S rRNA gene database and workbench compatible with ARB. Appl Environ Microbiol. 2006; 72:5069–72. 10.1128/AEM.03006-05 16820507 PMC1489311

[17] http://drive5.com/usearch/manual/uclust_algo.html.

[18] Rideout JR, He Y, Navas-Molina JA, Walters WA, Ursell LK, Gibbons SM, et al Subsampled open-reference clustering creates consistent, comprehensive OTU definitions and scales to billions of sequences. Peer J. 2014; 2:e545 10.7717/peerj.545 25177538 PMC4145071

[19] https://blast.ncbi.nlm.nih.gov/Blast.cgi?PROGRAM=blastn&PAGE_TYPE=BlastSearch&LINK_LOC=blasthome.

[20] metagenomeSeq. Statistical analysis for sparse high-throughput sequencing. http://cbcb.umd.edu/software/metagenomeSeq

[21] Dhariwal A, Chong J, Habib S, King IL, Agellon LB, Xia J. MicrobiomeAnalyst: a web-based tool for comprehensive statistical, visual and meta-analysis of microbiome data. Nucleic Acids Res. 2017; 45:W180–8. 10.1093/nar/gkx295 28449106 PMC5570177

[22] Walker DH, Paddock CD, Dumler JS. Emerging and re-emerging tick-transmitted rickettsial and ehrlichial infections. Med Clin North Am. 2008; 92:1345–61. 10.1016/j.mcna.2008.06.002 19061755

[23] Parola P, Paddock CD, Socolovschi C, Labruna MB, Mediannikov O, Kernif T, et al Update on tick-borne rickettsioses around the world: a geographic approach. Clin Microbiol Rev. 2013; 26:657–702. 10.1128/CMR.00032-13 24092850 PMC3811236

[24] Kernif T, Leulmi H, Raoult D, Parola P. Emerging tick-borne bacterial pathogens. Microbiol Spectr 2016; 4(3). 10.1128/microbiolspec.EI10-0012-2016 27337487

[25] Graham RMA, Donohue S, McMahon J, Jennison AV. Detection of Spotted Fever Group Rickettsia DNA by Deep Sequencing. Emerg Infect Dis. 2017; 23:1911–13. 10.3201/eid2311.170474 29048295 PMC5652451

[26] Gall CA, Reif KE, Scoles GA, Mason KL, Mousel M, Noh SM, et al The bacterial microbiome of *Dermacentor andersoni* ticks influences pathogen susceptibility. ISME J. 2016; 10:1846–55. 10.1038/ismej.2015.266 26882265 PMC5029153

[27] Estrada-Peña A, Cabezas-Cruz A. Towards the integrative analysis of tick microbiome. Ticks Tick Borne Dis. 2019; 10:34–35. 10.1016/j.ttbdis.2018.08.017 30196015

[28] Brouqui P, Parola P, Fournier PE, Raoult D. Spotted fever rickettsioses in southern and eastern Europe. FEMS Immunol Med Microbiol. 2007; 49:2–12. 10.1111/j.1574-695X.2006.00138.x 17266709

[29] Welinder-Olsson C, Kjellin E, Vaht K, Jacobsson S, Wenneras C. First case of human “*Candidatus* Neoehrlichia mikurensis” infection in a febrile patient with chronic lymphocytic leukemia. J Clin Microbiol. 2010; 48:1956–9. 10.1128/JCM.02423-09 20220155 PMC2863919

[30] Tijsse-Klasen E, Koopmans MP, Sprong H. Tick-borne pathogen—reversed and conventional discovery of disease. Front Public Health. 2014; 2:73 10.3389/fpubh.2014.00073 25072045 PMC4083466

[31] Portillo A, Santibáñez P, Palomar AM, Santibáñez S, Oteo JA. *'Candidatus* Neoehrlichia mikurensis' in Europe. New Microbes New Infect. 2018; 22:30–6. 10.1016/j.nmni.2017.12.011 29556406 PMC5857181

[32] Nyarko E, Grab DJ, Dumler JS. *Anaplasma phagocytophilum*-infected neutrophils enhance transmigration of *Borrelia burgdorferi* across the human blood brain barrier in vitro. Int J Parasitol. 2006; 36:601–5. 10.1016/j.ijpara.2006.01.014 16600247

[33] Grab DJ, Nyarko E, Barat NC, Nikolskaia OV, Dumler JS. *Anaplasma phagocytophilum-Borrelia burgdorferi* coinfection enhances chemokine, cytokine, and matrix metalloprotease expression by human brain microvascular endothelial cells. Clin Vaccine Immunol. 2007; 14:1420–4. 10.1128/CVI.00308-07 17898182 PMC2168173

[34] Carpi G, Cagnacci F, Wittekindt NE, Zhao F, Qi J, Tomsho LP, et al Metagenomic profile of the bacterial communities associated with *Ixodes ricinus* ticks. PLoS One. 2011; 6:e25604 10.1371/journal.pone.0025604 22022422 PMC3192763

[35] Nakao R, Abe T, Nijhof AM, Yamamoto S, Jongejan F, Ikemura T, et al A novel approach, based on BLSOMs (Batch Learning Self-Organizing Maps), to the microbiome analysis of ticks. ISME J. 2013; 7(5):1003–15. 10.1038/ismej.2012.171 23303373 PMC3635243

[36] Qiu Y, Nakao R, Ohnuma A, Kawamori F, Sugimoto C. Microbial population analysis of the salivary glands of ticks; a possible strategy for the surveillance of bacterial pathogens. PLoS One. 2014; 9:e103961 10.1371/journal.pone.0103961 25089898 PMC4121176

[37] René-Martellet M, Minard G, Massot R, Tran Van V, Valiente Moro C, Chabanne L, et al Bacterial microbiota associated with *Rhipicephalus sanguineus* (s.l.) ticks from France, Senegal and Arizona. Parasit Vectors. 2017; 10:416 10.1186/s13071-017-2352-9 28886749 PMC5591579

[38] Tekin S, Dowd SE, Davinic M, Bursali A, Keskin A. Pyrosequencing based assessment of bacterial diversity in Turkish *Rhipicephalus annulatus* and *Dermacentor marginatus* ticks (Acari: Ixodidae). Parasitol Res. 2017; 116:1055–1061. 10.1007/s00436-017-5387-0 28111714

[39] Estrada-Peña A, Cabezas-Cruz A, Pollet T, Vayssier-Taussat M, Cosson JF. High throughput sequencing and network analysis disentangle the microbial communities of ticks and hosts within and between ecosystems. Front Cell Infect Microbiol. 2018; 8:236 10.3389/fcimb.2018.00236 30038903 PMC6046413

[40] Werner JJ, Koren O, Hugenholtz P, DeSantis TZ, Walters WA, Capoaso JG, et al Impact of training sets on classification of high-throughput bacterial 16S rRNA gene surveys. ISME Journal. 2012; 6:94–103. 10.1038/ismej.2011.82 21716311 PMC3217155

[41] Sassera D, Beninati T, Bandi C, Bouman EA, Sacchi L, Fabbi M, et al *'Candidatus* Midichloria mitochondrii', an endosymbiont of the tick *Ixodes ricinus* with a unique intramitochondrial lifestyle. Int J Syst Evol Microbiol. 2006; 56:2535–40. 10.1099/ijs.0.64386-0 17082386

[42] Taylor M, Mediannikov O, Raoult D, Greub G. Endosymbiotic bacteria associated with nematodes, ticks and amoebae. FEMS Immunol Med Microbiol. 2012; 64:21–31. 10.1111/j.1574-695X.2011.00916.x 22126456

[43] Cisak E, Wójcik-Fatla A, Zając V, Sawczyn A, Sroka J, Dutkiewicz J. *Spiroplasma*—an emerging arthropod-borne pathogen? Ann Agric Environ Med. 2015; 22:589–93. 10.5604/12321966.1185758 26706960

[44] Mueller NJ, Tini GM, Weber A, Gaspert A, Husmann L, Bloemberg G, et al Hepatitis from *Spiroplasma* sp. in an immunocompromised patient. Am J Transplant. 2015; 15:2511–6. 10.1111/ajt.13254 25832127

[45] Van Treuren W, Ponnusamy L, Brinkerhoff RJ, Gonzalez A, Parobek CM, Juliano JJ, et al Variation in the microbiota of *Ixodes* ticks with regard to geography, species, and sex. Appl Environ Microbiol. 2015; 81:6200–9. 10.1128/AEM.01562-15 26150449 PMC4542252

[46] Randolph SE. Tick ecology: processes and patterns behind the epidemiological risk posed by ixodid ticks as vectors. Parasitology. 2004; 129:537–565. 15938504 10.1017/s0031182004004925

[47] Socolovschi C, Gaudart J, Bitam I, Huynh TP, Raoult D, Parola P. Why are there so few *Rickettsia conorii conorii*-infected *Rhipicephalus sanguineus* ticks in the wild? PLoS Negl Trop Dis. 2012; 6:e1697 10.1371/journal.pntd.0001697 22724035 PMC3378603

[48] Gilbert L, Aungier J, Tomkins JL. Climate of origin affects tick (*Ixodes ricinus*) host-seeking behavior in response to temperature: implications for resilience to climate change? Ecol Evol. 2014; 4:1186–98. 10.1002/ece3.1014 24772293 PMC3997332

[49] Greay TL, Gofton AW, Paparini A, Ryan UM, Oskam CL, Irwin PJ. Recent insights into the tick microbiome gained through next-generation sequencing. Parasit Vectors. 2018; 11:12 10.1186/s13071-017-2550-5 29301588 PMC5755153

[50] Diaz JH. Ticks, including tick paralysis *In* Bennett JE, Dolin R, Blaser MJ, editors. Mandell, Douglas, and Bennett’s principles and practice of infectious diseases 8th ed, vol 2 Philadelphia: Elsevier Saunders; 2015, p. 3266–3279.

