# Supplementary material for: Exploring the bacteriome in anthropophilic ticks: To investigate the vectors for diagnosis
Source: PLoS One. 2019 Mar 19;14(3):e0213384. doi: 10.1371/journal.pone.0213384 (PMC6424421; doi:10.1371/journal.pone.0213384)
Supplement: S1 Table — (DOC) [file pone.0213384.s001.doc]

Table S1: Details of the 99 samples analyzed in this study.

|  | **Tick species** | **Sample ID** | **Biosample accession number** | **Sex (or tick stage)** | **Sampling year** | **Location in La Rioja** | **Coordinates** | **Habitat** | **Sampling method** |
| --- | --- | --- | --- | --- | --- | --- | --- | --- | --- |
| 1. | *R. sanguineus* s.l. | 2GV15-22-A | SAMN10170891 | F | 2015 | San Vicente de la Sonsierra | 42º34'N;2º45'W | Scrubs; vegetation from trail borders | Direct capture |
| 2. | *R. sanguineus* s.l. | 2GV15-22-B | SAMN10170892 | F | 2015 | San Vicente de la Sonsierra | 42º34'N;2º45'W | Scrubs; vegetation from trail borders | Direct capture |
| 3. | *R. sanguineus* s.l. | 2GV15-22-C | SAMN10170893 | F | 2015 | San Vicente de la Sonsierra | 42º34'N;2º45'W | Scrubs; vegetation from trail borders | Direct capture |
| 4. | *R. sanguineus* s.l. | 2GV15-22-D | SAMN10170894 | F | 2015 | San Vicente de la Sonsierra | 42º34'N;2º45'W | Scrubs; vegetation from trail borders | Direct capture |
| 5. | *R. sanguineus* s.l. | 3GV12-81-B | SAMN10170895 | F | 2012 | Villalba de Rioja | 42º36'N; 2º53'W | Woodland, coniferous forest; vegetation from trail borders | Flagging |
| 6. | *R. sanguineus* s.l. | 3GV12-81-C | SAMN10170896 | F | 2012 | Villalba de Rioja | 42º36'N; 2º53'W | Woodland, coniferous forest; vegetation from trail borders | Flagging |
| 7. | *R. sanguineus* s.l. | 3GV12-81-D | SAMN10170897 | F | 2012 | Villalba de Rioja | 42º36'N; 2º53'W | Woodland, coniferous forest; vegetation from trail borders | Flagging |
| 8. | *R. sanguineus* s.l. | 3GV12-81-E | SAMN10170898 | F | 2012 | Villalba de Rioja | 42º36'N; 2º53'W | Woodland, coniferous forest; vegetation from trail borders | Flagging |
| 9. | *R. sanguineus* s.l. | 3GV12-81-G | SAMN10170899 | F | 2012 | Villalba de Rioja | 42º36'N; 2º53'W | Woodland, coniferous forest; vegetation from trail borders | Flagging |
| 10. | *R. sanguineus* s.l. | 3GV13-29-B | SAMN10170900 | F | 2013 | Villalba de Rioja | 42º36'N; 2º53'W | Woodland, coniferous forest; vegetation from trail borders | Flagging |
| 11. | *R. sanguineus* s.l. | 3GV13-35 | SAMN10170901 | F | 2013 | Soto de Cameros | 42º16'N;2º25'W | Scrubs; vegetation from trail borders | Direct capture |
| 12. | *R. sanguineus* s.l. | 2GV15-20-A | SAMN10170902 | M | 2015 | San Vicente de la Sonsierra | 42º34'N;2º45'W | Scrubs; vegetation from trail borders | Direct capture |
| 13. | *R. sanguineus* s.l. | 2GV15-20-C | SAMN10170903 | M | 2015 | San Vicente de la Sonsierra | 42º34'N;2º45'W | Scrubs; vegetation from trail borders | Direct capture |
| 14. | *R. sanguineus* s.l. | MM32 | SAMN10170904 | M | 2012 | Villalba de Rioja | 42º36'N; 2º53'W | Woodland, coniferous forest; vegetation from trail borders | Flagging |
| 15. | *R. sanguineus* s.l. | MM33 | SAMN10170905 | M | 2012 | Villalba de Rioja | 42º36'N; 2º53'W | Woodland, coniferous forest; vegetation from trail borders | Flagging |
| 16. | *R. sanguineus* s.l. | MM34 | SAMN10170906 | M | 2012 | Villalba de Rioja | 42º36'N; 2º53'W | Woodland, coniferous forest; vegetation from trail borders | Flagging |
| 17. | *R. sanguineus* s.l. | MM35 | SAMN10170907 | M | 2013 | Villamediana de Iregua | 42º25'N;2º25'W | Urban area, walls of a kennel | Direct capture |
| 18. | *R. sanguineus* s.l. | MM36 | SAMN10170908 | M | 2013 | Soto de Cameros | 42º16'N;2º25'W | Scrubs; vegetation from trail borders | Direct capture |
| 19. | *R. sanguineus* s.l. | MM37 | SAMN10170909 | M | 2013 | Villamediana de Iregua | 42º25'N;2º25'W | Urban area, walls of a kennel | Direct capture |
| 20. | *R. sanguineus* s.l. | MM38 | SAMN10170910 | M | 2013 | Villamediana de Iregua | 42º25'N;2º25'W | Urban area, walls of a kennel | Direct capture |
| 21. | *R. sanguineus* s.l. | MM39 | SAMN10170911 | M | 2013 | Villamediana de Iregua | 42º25'N;2º25'W | Urban area, walls of a kennel | Direct capture |
| 22. | *R. sanguineus* s.l. | MM40 | SAMN10170912 | M | 2013 | Villamediana de Iregua | 42º25'N;2º25'W | Urban area, walls of a kennel | Direct capture |
| *23.* | *H. punctata* | 3GV14-2-A | SAMN10170913 | F | 2014 | Villalba de Rioja | 42º36'N; 2º53'W | Woodland, coniferous forest; vegetation from trail borders | Flagging |
| 24. | *H. punctata* | 3GV14-2-B | SAMN10170914 | F | 2014 | Villalba de Rioja | 42º36'N; 2º53'W | Woodland, coniferous forest; vegetation from trail borders | Flagging |
| 25. | *H. punctata* | 3GV14-2-C | SAMN10170915 | F | 2014 | Villalba de Rioja | 42º36'N; 2º53'W | Woodland, coniferous forest; vegetation from trail borders | Flagging |
| 26. | *H. punctata* | 3GV14-2-D | SAMN10170916 | F | 2014 | Villalba de Rioja | 42º36'N; 2º53'W | Woodland, coniferous forest; vegetation from trail borders | Flagging |
| 27. | *H. punctata* | 3GV14-2-E | SAMN10170917 | F | 2014 | Villalba de Rioja | 42º36'N; 2º53'W | Woodland, coniferous forest; vegetation from trail borders | Flagging |
| 28. | *H. punctata* | 2GV13-5-A | SAMN10170918 | F | 2013 | Villalba de Rioja | 42º36'N; 2º53'W | Woodland, coniferous forest; vegetation from trail borders | Flagging |
| 29. | *H. punctata* | 2GV13-5-B | SAMN10170919 | F | 2013 | Villalba de Rioja | 42º36'N; 2º53'W | Woodland, coniferous forest; vegetation from trail borders | Flagging |
| 30. | *H. punctata* | 2GV13-5-C | SAMN10170920 | F | 2013 | Villalba de Rioja | 42º36'N; 2º53'W | Woodland, coniferous forest; vegetation from trail borders | Flagging |
| 31. | *H. punctata* | 2GV13-5-D | SAMN10170921 | F | 2013 | Villalba de Rioja | 42º36'N; 2º53'W | Woodland, coniferous forest; vegetation from trail borders | Flagging |
| 32. | *H. punctata* | 2GV13-5-E | SAMN10170922 | F | 2013 | Villalba de Rioja | 42º36'N; 2º53'W | Woodland, coniferous forest; vegetation from trail borders | Flagging |
| 33. | *H. punctata* | 2GV13-5-F | SAMN10170923 | F | 2013 | Villalba de Rioja | 42º36'N; 2º53'W | Woodland, coniferous forest; vegetation from trail borders | Flagging |
| 34. | *H. punctata* | 2GV13-5-G | SAMN10170924 | F | 2013 | Villalba de Rioja | 42º36'N; 2º53'W | Woodland, coniferous forest; vegetation from trail borders | Flagging |
| 35. | *H. punctata* | 2GV13-5-H | SAMN10170925 | F | 2013 | Villalba de Rioja | 42º36'N; 2º53'W | Woodland, coniferous forest; vegetation from trail borders | Flagging |
| 36. | *H. punctata* | 2GV13-5-I | SAMN10170926 | F | 2013 | Villalba de Rioja | 42º36'N; 2º53'W | Woodland, coniferous forest; vegetation from trail borders | Flagging |
| 37. | *H. punctata* | 2GV13-5-J | SAMN10170927 | F | 2013 | Villalba de Rioja | 42º36'N; 2º53'W | Woodland, coniferous forest; vegetation from trail borders | Flagging |
| 38. | *H. punctata* | 1GV14-70-A | SAMN10170928 | M | 2014 | Villalba de Rioja | 42º36'N; 2º53'W | Woodland, coniferous forest; vegetation from trail borders | Flagging |
| 39. | *H. punctata* | 1GV14-70-C | SAMN10170929 | M | 2014 | Villalba de Rioja | 42º36'N; 2º53'W | Woodland, coniferous forest; vegetation from trail borders | Flagging |
| 40. | *H. punctata* | 1GV14-70-D | SAMN10170930 | M | 2014 | Villalba de Rioja | 42º36'N; 2º53'W | Woodland, coniferous forest; vegetation from trail borders | Flagging |
| 41. | *H. punctata* | 1GV14-70-E | SAMN10170931 | M | 2014 | Villalba de Rioja | 42º36'N; 2º53'W | Woodland, coniferous forest; vegetation from trail borders | Flagging |
| 42. | *H. punctata* | MM12 | SAMN10170932 | M | 2010 | Villalba de Rioja | 42º36'N; 2º53'W | Woodland, coniferous forest; vegetation from trail borders | Flagging |
| 43. | *H. punctata* | MM14 | SAMN10170933 | M | 2010 | Villalba de Rioja | 42º36'N; 2º53'W | Woodland, coniferous forest; vegetation from trail borders | Flagging |
| 44. | *H. punctata* | MM15 | SAMN10170934 | M | 2010 | Villalba de Rioja | 42º36'N; 2º53'W | Woodland, coniferous forest; vegetation from trail borders | Flagging |
| 45. | *H. punctata* | MM16 | SAMN10170935 | M | 2010 | Almarza | 42º14'N; 2º35'W | Woodland, broad-leaved forest; vegetation from trail borders | Flagging |
| 46. | *H. punctata* | MM17 | SAMN10170936 | M | 2010 | Tobía | 42º17'N; 2º58'W | Scrubs; vegetation from trail borders | Direct capture |
| 47. | *H. punctata* | MM18 | SAMN10170937 | M | 2010 | Tobía | 42º17'N; 2º58'W | Scrubs; vegetation from trail borders | Direct capture |
| 48. | *H. punctata* | MM19 | SAMN10170938 | M | 2010 | Tobía | 42º17'N; 2º58'W | Scrubs; vegetation from trail borders | Direct capture |
| 49. | *H. punctata* | MM20 | SAMN10170939 | M | 2010 | Enciso | 42º8'N; 2º16'W | Natural grasslands; vegetation from trail borders | Direct capture |
| *50.* | *D. marginatus* | 2GV14-1 | SAMN10170940 | F | 2014 | Villalba de Rioja | 42º36'N; 2º53'W | Woodland, coniferous forest; vegetation from trail borders | Direct capture |
| 51. | *D. marginatus* | 2GV14-11 | SAMN10170941 | F | 2014 | Villalba de Rioja | 42º36'N; 2º53'W | Woodland, coniferous forest; vegetation from trail borders | Direct capture |
| 52. | *D. marginatus* | 2GV14-12 | SAMN10170942 | F | 2014 | Villalba de Rioja | 42º36'N; 2º53'W | Woodland, coniferous forest; vegetation from trail borders | Direct capture |
| 53. | *D. marginatus* | 2GV14-14 | SAMN10170943 | F | 2014 | Villalba de Rioja | 42º36'N; 2º53'W | Woodland, coniferous forest; vegetation from trail borders | Direct capture |
| 54. | *D. marginatus* | 2GV14-15 | SAMN10170944 | F | 2014 | Villalba de Rioja | 42º36'N; 2º53'W | Woodland, coniferous forest; vegetation from trail borders | Direct capture |
| 55. | *D. marginatus* | 2GV14-16 | SAMN10170945 | F | 2014 | Villalba de Rioja | 42º36'N; 2º53'W | Woodland, coniferous forest; vegetation from trail borders | Direct capture |
| 56. | *D. marginatus* | 2GV14-2 | SAMN10170946 | F | 2014 | Villalba de Rioja | 42º36'N; 2º53'W | Woodland, coniferous forest; vegetation from trail borders | Direct capture |
| 57. | *D. marginatus* | 2GV14-3 | SAMN10170947 | F | 2014 | Villalba de Rioja | 42º36'N; 2º53'W | Woodland, coniferous forest; vegetation from trail borders | Direct capture |
| 58. | *D. marginatus* | 2GV14-53 | SAMN10170948 | F | 2014 | Villalba de Rioja | 42º36'N; 2º53'W | Woodland, coniferous forest; vegetation from trail borders | Direct capture |
| 59. | *D. marginatus* | 2GV14-54 | SAMN10170949 | F | 2014 | Villalba de Rioja | 42º36'N; 2º53'W | Woodland, coniferous forest; vegetation from trail borders | Direct capture |
| 60. | *D. marginatus* | 2GV14-55 | SAMN10170950 | F | 2014 | Villalba de Rioja | 42º36'N; 2º53'W | Woodland, coniferous forest; vegetation from trail borders | Direct capture |
| 61. | *D. marginatus* | 2GV14-56 | SAMN10170951 | F | 2014 | Villalba de Rioja | 42º36'N; 2º53'W | Woodland, coniferous forest; vegetation from trail borders | Direct capture |
| 62. | *D. marginatus* | 2GV14-57 | SAMN10170952 | F | 2014 | Villalba de Rioja | 42º36'N; 2º53'W | Woodland, coniferous forest; vegetation from trail borders | Direct capture |
| 63. | *D. marginatus* | 2GV14-58 | SAMN10170953 | F | 2014 | Villalba de Rioja | 42º36'N; 2º53'W | Woodland, coniferous forest; vegetation from trail borders | Direct capture |
| 64. | *D. marginatus* | 2GV14-59 | SAMN10170954 | F | 2014 | Villalba de Rioja | 42º36'N; 2º53'W | Woodland, coniferous forest; vegetation from trail borders | Direct capture |
| 65. | *D. marginatus* | 2GV14-60 | SAMN10170955 | F | 2014 | Villalba de Rioja | 42º36'N; 2º53'W | Woodland, coniferous forest; vegetation from trail borders | Direct capture |
| 66. | *D. marginatus* | 2GV14-61 | SAMN10170956 | F | 2014 | Villalba de Rioja | 42º36'N; 2º53'W | Woodland, coniferous forest; vegetation from trail borders | Direct capture |
| 67. | *D. marginatus* | 2GV14-62 | SAMN10170957 | F | 2014 | Villalba de Rioja | 42º36'N; 2º53'W | Woodland, coniferous forest; vegetation from trail borders | Direct capture |
| 68. | *D. marginatus* | 2GV14-8 | SAMN10170958 | F | 2014 | Villalba de Rioja | 42º36'N; 2º53'W | Woodland, coniferous forest; vegetation from trail borders | Direct capture |
| 69. | *D. marginatus* | 2GV14-9 | SAMN10170959 | F | 2014 | Villalba de Rioja | 42º36'N; 2º53'W | Woodland, coniferous forest; vegetation from trail borders | Direct capture |
| 70. | *D. marginatus* | MM21 | SAMN10170960 | M | 2010 | Daroca de Rioja | 42º22'N; 2º35'W | Woodland, mixed forest; vegetation from trail borders | Direct capture |
| 71. | *D. marginatus* | MM22 | SAMN10170961 | M | 2010 | Daroca de Rioja | 42º22'N; 2º35'W | Woodland, mixed forest; vegetation from trail borders | Direct capture |
| 72. | *D. marginatus* | MM23 | SAMN10170962 | M | 2010 | Daroca de Rioja | 42º22'N; 2º35'W | Woodland, mixed forest; vegetation from trail borders | Direct capture |
| 73. | *D. marginatus* | MM24 | SAMN10170963 | M | 2010 | Daroca de Rioja | 42º22'N; 2º35'W | Woodland, mixed forest; vegetation from trail borders | Direct capture |
| 74. | *D. marginatus* | MM25 | SAMN10170964 | M | 2010 | Villalba de Rioja | 42º36'N; 2º53'W | Woodland, coniferous forest; vegetation from trail borders | Direct capture |
| 75. | *D. marginatus* | MM26 | SAMN10170965 | M | 2010 | Villalba de Rioja | 42º36'N; 2º53'W | Woodland, coniferous forest; vegetation from trail borders | Direct capture |
| 76. | *D. marginatus* | MM27 | SAMN10170966 | M | 2010 | Villalba de Rioja | 42º36'N; 2º53'W | Woodland, coniferous forest; vegetation from trail borders | Direct capture |
| 77. | *D. marginatus* | MM28 | SAMN10170967 | M | 2010 | Villalba de Rioja | 42º36'N; 2º53'W | Woodland, coniferous forest; vegetation from trail borders | Direct capture |
| 78. | *D. marginatus* | MM29 | SAMN10170968 | M | 2010 | Villalba de Rioja | 42º36'N; 2º53'W | Woodland, coniferous forest; vegetation from trail borders | Direct capture |
| 79. | *D. marginatus* | MM30 | SAMN10170969 | M | 2010 | Villalba de Rioja | 42º36'N; 2º53'W | Woodland, coniferous forest; vegetation from trail borders | Direct capture |
| *80.* | *I. ricinus* | 3GV14-4-A | SAMN10170970 | F | 2014 | Villalba de Rioja | 42º36'N; 2º53'W | Woodland, coniferous forest; vegetation from trail borders | Flagging |
| 81. | *I. ricinus* | 3GV14-4-B | SAMN10170971 | F | 2014 | Villalba de Rioja | 42º36'N; 2º53'W | Woodland, coniferous forest; vegetation from trail borders | Flagging |
| 82. | *I. ricinus* | 3GV14-4-C | SAMN10170972 | F | 2014 | Villalba de Rioja | 42º36'N; 2º53'W | Woodland, coniferous forest; vegetation from trail borders | Flagging |
| 83. | *I. ricinus* | 3GV14-4-D | SAMN10170973 | F | 2014 | Villalba de Rioja | 42º36'N; 2º53'W | Woodland, coniferous forest; vegetation from trail borders | Flagging |
| 84. | *I. ricinus* | 3GV14-4-E | SAMN10170974 | F | 2014 | Villalba de Rioja | 42º36'N; 2º53'W | Woodland, coniferous forest; vegetation from trail borders | Flagging |
| 85. | *I. ricinus* | 3GV14-9-C | SAMN10170975 | F | 2014 | Torrecilla en Cameros | 42º16'N; 2º35'W | Scrubs; vegetation from trail borders | Direct capture |
| 86. | *I. ricinus* | 3GV14-9-J | SAMN10170976 | F | 2014 | Torrecilla en Cameros | 42º16'N; 2º35'W | Scrubs; vegetation from trail borders | Direct capture |
| 87. | *I. ricinus* | MM3* | SAMN10170977 | M | 2010 | Lumbreras | 42º8'N; 2º33'W | Woodland, coniferous forest; vegetation from trail borders | Direct capture |
| 88. | *I. ricinus* | NM14** | SAMN10170978 | N | 2010 | Almarza | 42º14'N; 2º35'W | Woodland, broad-leaved forest; vegetation from trail borders | Flagging |
| 89. | *I. ricinus* | NM15** | SAMN10170979 | N | 2010 | Almarza | 42º14'N; 2º35'W | Woodland, broad-leaved forest; vegetation from trail borders | Flagging |
| 90. | *I. ricinus* | NM16** | SAMN10170980 | N | 2010 | Almarza | 42º14'N; 2º35'W | Woodland, broad-leaved forest; vegetation from trail borders | Flagging |
| 91. | *I. ricinus* | NM17** | SAMN10170981 | N | 2010 | Almarza | 42º14'N; 2º35'W | Woodland, broad-leaved forest; vegetation from trail borders | Flagging |
| 92. | *I. ricinus* | NM18** | SAMN10170982 | N | 2010 | Almarza | 42º14'N; 2º35'W | Woodland, broad-leaved forest; vegetation from trail borders | Flagging |
| 93. | *I. ricinus* | NM19** | SAMN10170983 | N | 2010 | Almarza | 42º14'N; 2º35'W | Woodland, broad-leaved forest; vegetation from trail borders | Flagging |
| 94. | *I. ricinus* | NM20** | SAMN10170984 | N | 2010 | Almarza | 42º14'N; 2º35'W | Woodland, broad-leaved forest; vegetation from trail borders | Flagging |
| 95. | *I. ricinus* | 4GV14-33-F** | SAMN10170985 | N | 2014 | Tobía | 42º15'N; 2º53'W | Woodland, broad-leaved forest; underwood vegetation. | Flagging |
| 96. | *I. ricinus* | 4GV14-33-G** | SAMN10170986 | N | 2014 | Tobía | 42º15'N; 2º53'W | Woodland, broad-leaved forest; underwood vegetation. | Flagging |
| 97. | *I. ricinus* | 4GV14-34-A** | SAMN10170987 | N | 2014 | Tobía | 42º15'N; 2º53'W | Woodland, broad-leaved forest; underwood vegetation. | Flagging |
| 98. | *I. ricinus* | 4GV14-34-B** | SAMN10170988 | N | 2014 | Tobía | 42º15'N; 2º53'W | Woodland, broad-leaved forest; underwood vegetation. | Flagging |
| 99. | *I. ricinus* | 1GV14-19** | SAMN10170989 | N | 2014 | Villalba de Rioja | 42º36'N; 2º53'W | Woodland, coniferous forest; vegetation from trail borders | Flagging |

*R. sanguineus* s.l.: *Rhipicephalus sanguineus* sensu lato; *H. punctata*: *Haemaphysalis punctata*; *D. marginatus*: *Dermacentor marginatus*; *I. ricinus*: *Ixodes ricinus*; F: female; M: Male; N: nymphs; *Pool of two half ticks; ** Pool of ten nymphs. All samples belong to the Bioproject accession number PRJNA494526 from the NCBI Sequence Read Archive (SRA) database.
