# Supplementary figures and images for: Exploring the bacteriome in anthropophilic ticks: To investigate the vectors for diagnosis
Source: PLoS One. 2019 Mar 19;14(3):e0213384. doi: 10.1371/journal.pone.0213384 (PMC6424421; doi:10.1371/journal.pone.0213384)

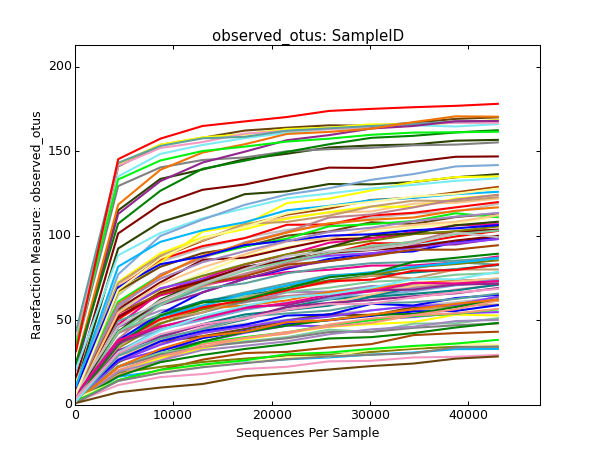

Supplement: S1 Fig — (TIFF) [file pone.0213384.s006.tiff]

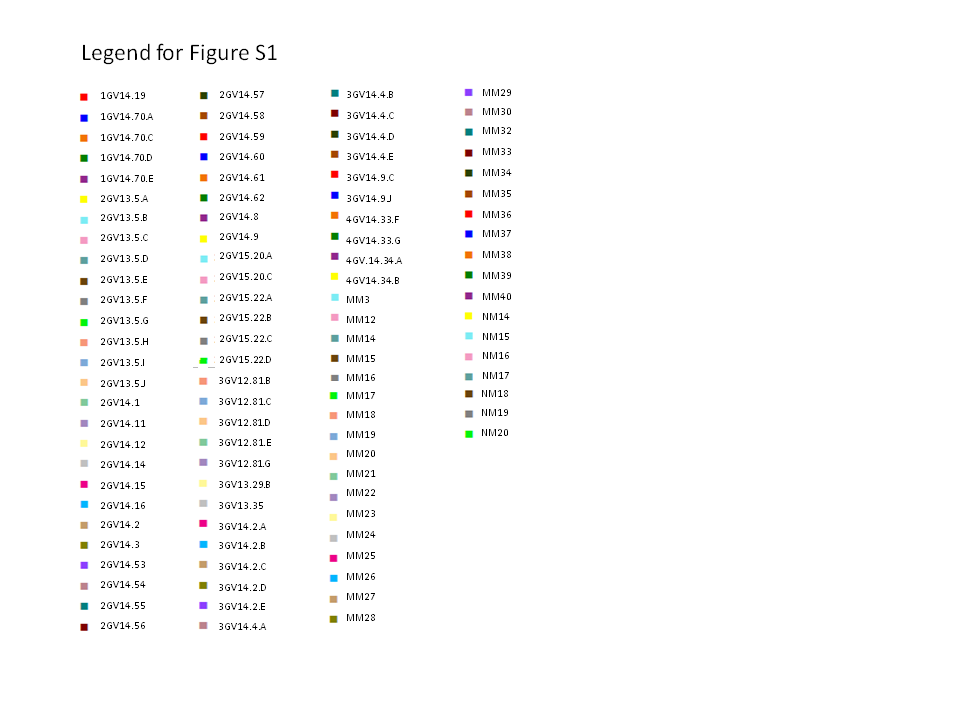

Supplement: S2 Fig — Sample IDs included in this study. (TIF) [file pone.0213384.s007.tif]

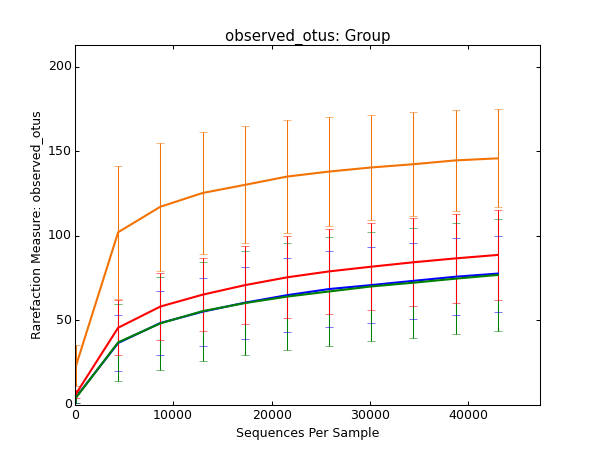

Supplement: S3 Fig — (TIFF) [file pone.0213384.s008.tiff]

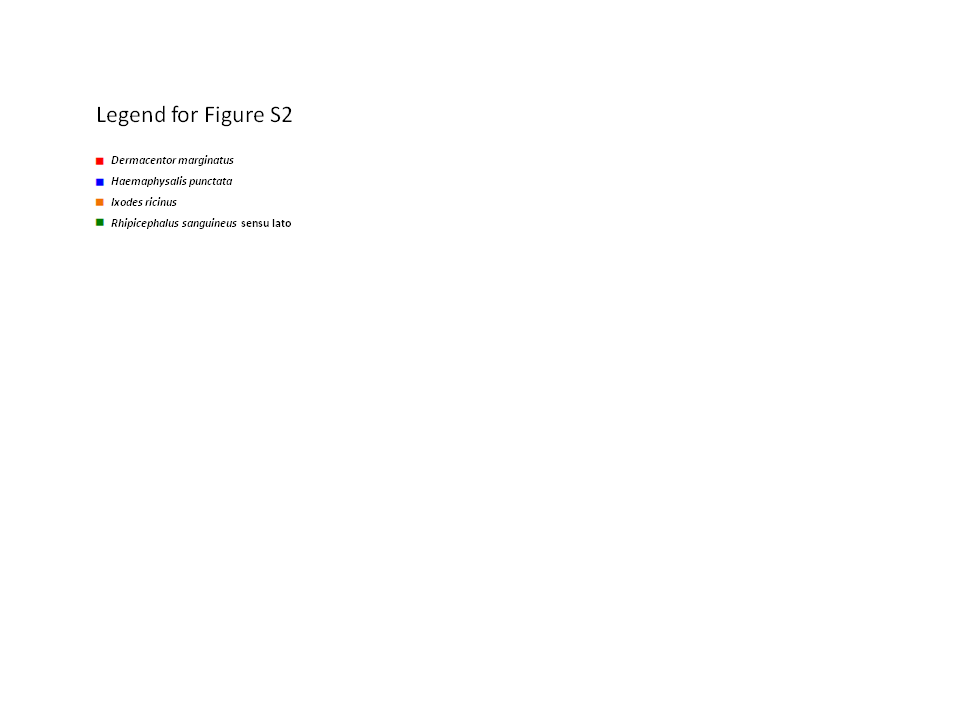

Supplement: S4 Fig — Sample groups (tick species) included in this study. (TIF) [file pone.0213384.s009.tif]
